# Supramolecular FRET photocyclodimerization of anthracenecarboxylate with naphthalene-capped γ-cyclodextrin

**DOI:** 10.3762/bjoc.7.38

**Published:** 2011-03-07

**Authors:** Qian Wang, Cheng Yang, Gaku Fukuhara, Tadashi Mori, Yu Liu, Yoshihisa Inoue

**Affiliations:** 1 Department of Applied Chemistry, Osaka University, 2-1 Yamada-oka, Suita 565-0871, Japan; 2 Department of Chemistry and State Key Laboratory of Elemento-Organic Chemistry, Nankai University Tianjin, 300071 (China)

**Keywords:** anthracenecarboxylic acid, capped γ-cyclodextrin, FRET sensitization, photochirogenesis, photocyclodimerization

## Abstract

γ-Cyclodextrin (CD) derivatives with a naphthalene moiety anchored to one or two of the glucose units of the CD were synthesized in order to investigate the effects of flexible and rigid capping upon complexation, as well as Förster resonance energy transfer (FRET) and photochirogenic behavior of anthracenecarboxylate (AC) moieties. UV–vis, circular dichroism and fluorescence spectral studies revealed that two AC molecules are simultaneously included in the modified γ-CD cavity to form a right-handed screw and also that the naphthalene cap efficiently transfers the singlet energy to AC included in the CD cavity via the FRET mechanism. Compared to native γ-CD, the modified γ-CDs showed much higher first association constants (*K*_1_) but relatively lower second association constants (*K*_2_) for AC, leading to two-fold larger overall affinities (*K*_1_*K*_2_). Photocyclodimerization of AC with these modified γ-CDs produced more head-to-head (HH) dimers in much better enantiomeric excesses (ee) for *anti*-HH dimer compared to native γ-CD. Interestingly, FRET excitation further enhanced the chemical and optical yields of *anti*-HH dimer up to 36% and 35% ee, for which the highly efficient FRET sensitization within the CD cavity, minimizing the “contamination” from the achiral “outside” photoreaction, is responsible. FRET sensitization also enabled us to achieve the catalytic photocyclodimerization of AC with a sub-equivalent amount of chiral supramolecular host.

## Introduction

Chiral photochemistry, often characterized by low optical yields, remains a great challenge for chemists [1–3]. This situation reflects primarily the difficulty in efficiently transferring the stereochemical information of the chiral source to the substrate in the electronically excited state. Thus, a supramolecular approach in chiral photochemistry could be a promising strategy for critically controlling the stereochemical outcome via intimate, long-lasting contacts with the photosubstrate through non-covalent interactions in the ground state [4–11]. The geometrical and functional complementarity and the subsequent induced fit between chiral host and guest substrate should play a crucial role in determining the stereochemical fate of chiral photoreaction, and therefore the design of chiral host is considered to be one of the most important aspects for manipulating stereoselectivity in supramolecular photochirogenesis.

**Scheme 1:**
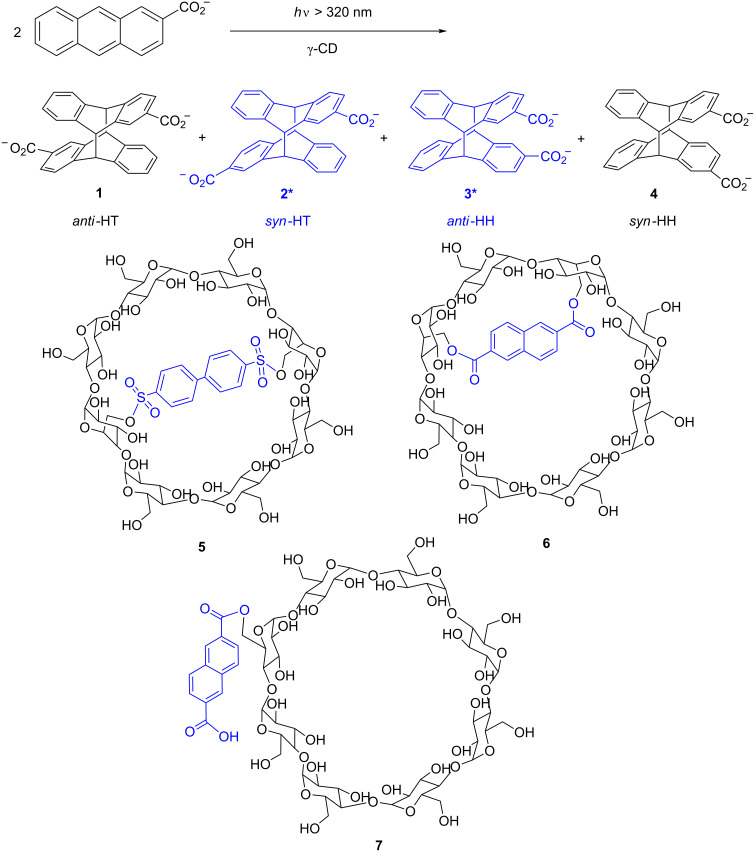
Biphenyl-capped (**5**), naphthalene-capped (**6**), and naphthalene-appended γ-cyclodextrin (**7**).

We have recently focused our attention on enantiodifferentiation in the photocyclodimerization of anthracenecarboxylate (AC) as a representative bimolecular photochirogenic system for the elucidation of the factors and mechanism that control supramolecular photochirogenesis [12–22]. Photocyclodimerization of AC leads to the formation of four stereoisomeric cyclodimers **1–4**, of which *syn-*head-to-tail (HT) **2** and *anti-*head-to-head (HH) **3** are chiral (Scheme 1). This chiral photoreaction turned out to be an ideal benchmark system for exploring and comparing the performance of various chiral hosts, such as cyclodextrins (CDs), proteins and chiral hydrogen-bonding templates. γ-CD can significantly accelerate the photocyclodimerization of AC by forming a 1:2 host–guest complex with AC in aqueous solutions [12]. Altering the chiral environment of γ-CD by rim modification is a convenient, yet effective, tool for manipulating the stereoselectivity of AC photocyclodimerization. In our previous studies, we have shown that a capping modification of γ-CD causes a dramatic switching of stereoselectivity in AC photodimerization [12,22]. Thus, by using native γ-CD the chiral cyclodimer **2** is obtained in 41% enantiomeric excess (ee), whereas biphenyl-capped γ-CD **5** yields antipodal **2** in −57% ee (Scheme 1) [22]: Where the positive/negative sign of ee indicates a higher yield of first/second eluted enantiomer detected on the chiral HPLC analysis. These observations prompted us to design and synthesize a more rigidly capped 6^A^,6^C^-(2,6-naphthalenedicarboxyl)-γ-CD **6**. This modification further enabled us to trigger the photocyclodimerization via Förster resonance energy transfer (FRET) sensitization.

## Results and Discussion

Naphthalene-capped γ-CD **6** was synthesized by the reaction of 6^A^,6^C^-ditosyl-γ-CD [23] with disodium 2,6-naphthalenedicarboxylate in DMSO. An attempt to synthesize the regioisomeric 6^A^,6^D^-(2,6-naphthalenedicarboxyl)-γ-CD by reacting 6^A^,6^D^-ditosyl-γ-CD with disodium 2,6-naphthalenedicarboxylate was unsuccessful, presumably due to the longer distance between the A and D glucose units to be bridged by the 2,6-naphthalenedicarboxylate unit. For the purposes of comparison, naphthalene-appended γ-CD **7** was synthesized by reacting γ-CD with 2,6-naphthalenedicarboxylic acid. Modified γ-CD **7** was found to be sparingly soluble in water, probably due to the intermolecular aggregation of **7** by successive penetration of the naphthalene moiety into the cavity of another CD. The solubility of **7** was significantly enhanced by adding sodium carbonate. In contrast, capped γ-CD **6** showed a much higher solubility in water, as the naphthalene cap can hardly interact with another CD.

**Figure 1:**
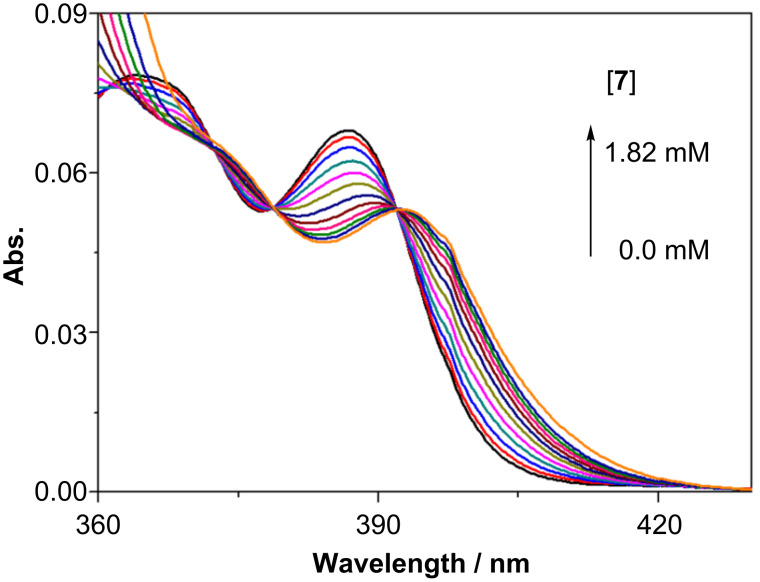
UV–vis spectral changes of 0.2 mM AC upon increasing the concentration of **7** in pH 9 phosphate buffer at 25 °C.

The complexation behavior of AC with modified γ-CDs **6** and **7** was investigated by UV–vis, circular dichroism and fluorescence spectral studies. As shown in Figure 1, the addition of **7** to an aqueous solution of AC (0.2 mM) caused an evident bathochromic shift of the ^1^*L*_a_ band of AC, which is probably due to the stacking complexation of two AC molecules in a single γ-CD cavity.

**Figure 2:**
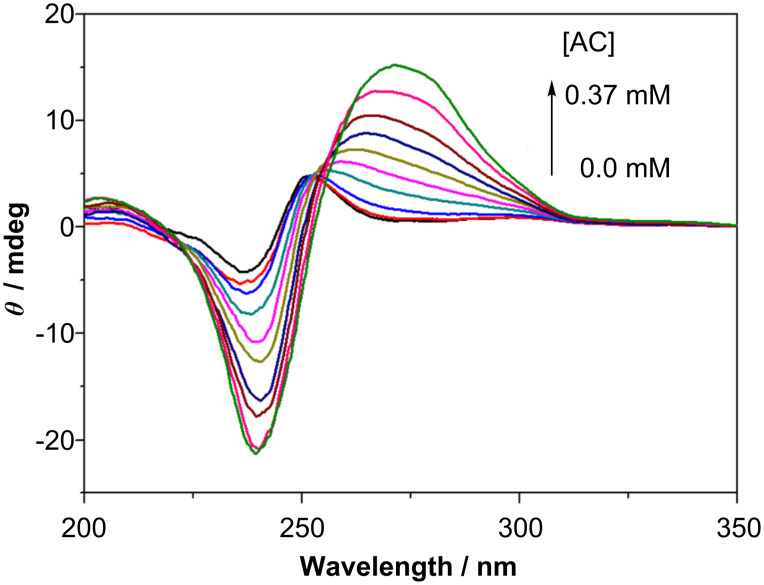
Circular dichroism spectra of **7** (0.2 mM) in the presence of 0, 0.0083, 0.025, 0.048, 0.071, 0.093, 0.13, 0.16, 0.22, 0.27 and 0.37 mM AC in pH 9 phosphate buffer at 20 °C.

**Figure 3:**
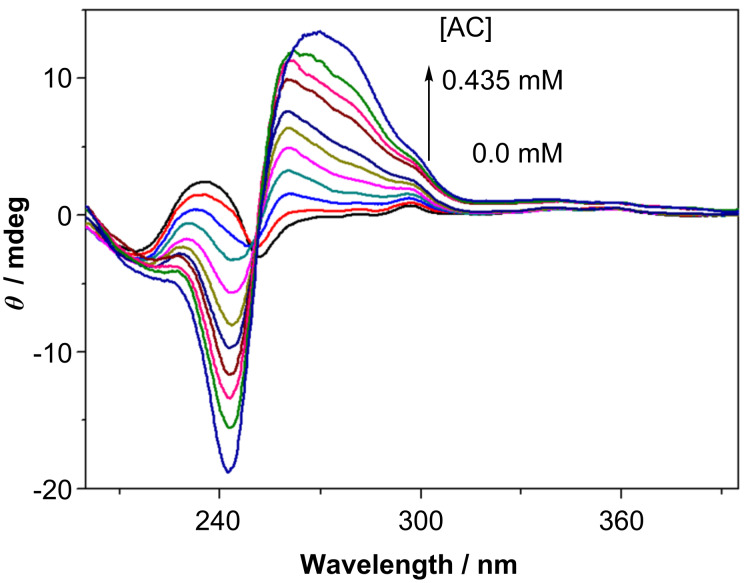
Circular dichroism spectra of **6** (0.2 mM) in the presence of 0, 0.0083, 0.025, 0.048, 0.071, 0.093, 0.11, 0.15, 0.18, 0.21 and 0.26 mM AC in pH 9 phosphate buffer at 20 °C.

Modified γ-CDs **6** and **7** showed moderate circular dichroism signals in the naphthalene-absorbing region. As shown in Figure 2, naphthalene-appended γ-CD **7** at 0.2 mM concentration gave a bisignate circular dichroism signal even in the absence of AC. As the intensity (Δε) decreased at lower concentrations, this bisignate signal is thought to be a real exciton couplet arising from the self-aggregation of **7**, where the included naphthalene chromophores are arranged in a right-handed screw [24]. As can be seen from Figure 3, naphthalene-capped γ-CD **6** gave only weak induced circular dichroism signals at the ^1^*L*_a_ and ^1^*B*_b_ bands of the naphthalene chromophore, the intensity of which was not concentration-dependent.

The addition of AC (0–0.26 mM) to the above solutions of **7** or **6** (0.2 mM) produced a strong positive exciton couplet at the ^1^*B*_b_ transition of AC (Figure 2 and Figure 3) which overwhelmed the inherent signals. The positive couplet observed indicates the right-handed helical conformation of the two AC molecules in the γ-CD cavity [24].

**Figure 4:**
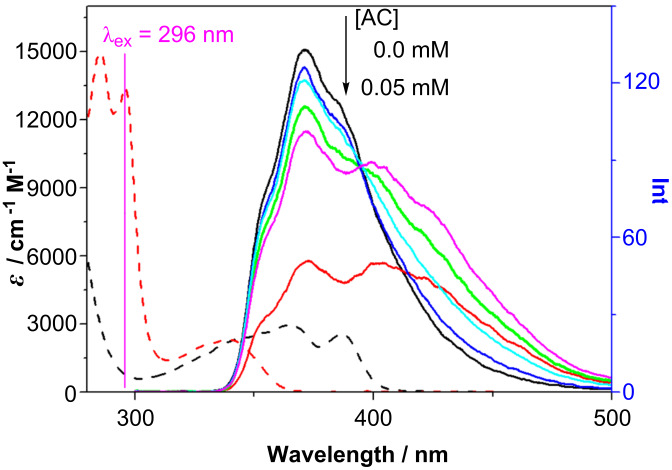
UV–vis spectra of AC (black dashed line) and **7** (red dashed line) and fluorescence spectra of **7** (0.02 mM) in the presence of 0.0, 0.0041, 0.0125, 0.025, 0.0375 and 0.05 mM AC in pH 9 phosphate buffer at 20 °C. λ_ex_ = 296 nm.

Fluorescence spectral behavior was examined at a lower host concentration in order to observe the fluorescence from both the 1:1 and 1:2 complexes with AC. Upon the addition of AC (0–0.05 mM) to a solution of **7** (0.02 mM), the naphthalene fluorescence at 373 nm was gradually reduced in intensity with an accompanying increase of AC fluorescence at 426 nm (Figure 4). The reduction of fluorescence intensity amounted to 27% at an AC concentration of 0.0375 mM, even although the absorbance of 0.0375 mM AC is only 11% of 0.02 mM naphthalene at the excitation wavelength (296 nm) and the internal filter effect of AC at 350–400 nm is negligible (absorbance <0.1). Considering the nice spectral overlap of the naphthalene fluorescence with the AC absorption (Figure 4), we conclude that Förster resonance energy transfer (FRET) is operating from naphthalene-appended **7** to AC residing in the cavity. Further addition of AC up to 0.05 mM caused a global decrease of the fluorescence of both **7** and AC, which can be rationalized by the increased formation of a 1:2 complex at the higher AC concentration, leading to efficient FRET and photocyclodimerization in the cavity.

Nonlinear least-squares fits of the UV–vis spectral titration data to the stepwise 1:2 complexation model gave binding constants for each step: *K*_1_ = 1050 M^−1^ and *K*_2_ = 18600 M^−1^ for **6** and *K*_1_ = 620 M^−1^ and *K*_2_ = 22300 M^−1^ for **7** at 25 °C. When compared to the corresponding values reported for native γ-CD (*K*_1_ = 182 M^−1^ and *K*_2_ = 56700 M^−1^) [12], the first binding constant was enhanced by a factor of 3.5–5.8 by introducing the naphthalene moiety, whilst the second binding constant was reduced by a factor of 2.5–3.0, thus making the overall binding constant (*K*_1_*K*_2_) approximately twice as high. It is known that the small *K*_1_ for native γ-CD is due to the oversized cavity which cannot provide tight contacts for a single AC molecule. Only when a second AC is introduced into the same cavity, are close contacts possible for two AC molecules with γ-CD walls thus leading to a much larger *K*_2_. We attribute the enhanced *K*_1_ values for **6** and **7** to the increased hydrophobicity of the naphthalene-modified γ-CD cavity. On the contrary, the naphthalene moieties reduce the *K*_2_ values, probably due to steric hindrance and the restricted conformation and orientation available for ACs in the cavity. The higher overall affinities (*K*_1_*K*_2_) for **6** and **7** than for native γ-CD indicate the positive effect of aromatic modification on AC complexation.

An aqueous buffer solution (pH 9) of AC (0.4 mM) and modified γ-CD (2 mM) was photoirradiated at 360 nm with a Xenon lamp fitted with a band-pass filter. The product distribution and the ee of chiral photodimers, both determined by chiral HPLC, are shown in Table 1. As a general tendency, the HH dimers were preferred by introducing the aromatic substituents. Thus, the HH/HT ratio was dramatically enhanced from 0.12 for native γ-CD to 0.4–1.0 for biphenyl- or naphthalene-modified γ-CDs **5**–**7**. In particular, the use of the capped γ-CD **6** led to the preferential formation of chiral **3** in 34% yield at 0 °C. The increased hydrophobicity around the primary rim of these modified γ-CDs favors inclusion of the non-polar aromatic part of AC near the primary portal and the polar carboxylate part near the secondary portal to give the HH-oriented precursor complex. In contrast, the HT-oriented precursor complex, in which one of AC’s carboxylate is positioned at the primary rim, should be less stable due to increased hydrophobicity, leading to the switching of product population to HH dimers.

**Table 1:** Photocyclodimerization of 2-anthracenecarboxylate (AC) in the presence of native and modified γ-cyclodextrins (CDs)^a^*.*

host	λ/nm^b^	CD/AC^c^	*T*/°C	relative yield/%^d^				HH/HT^e^	*anti/syn*		ee/%^d,f^	
		
				**1**	**2**	**3**	**4**		**1**/**2**	**3**/**4**	**2**	**3**

γ-CD^g^	365	5	0	43	46	6	5	0.12	0.9	1.2	41	−1
**5**^h^	365	5	0	39	28	20	13	0.49	1.4	1.5	−58	−14
**6**	360	5	35	40	30	23	7	0.43	1.3	3.3	16	18
		5	20	38	29	25	9	0.51	1.3	2.8	20	21
		5	10	34	25	30	11	0.69	1.4	2.7	23	25
		5	0	29	23	34	13	0.90	1.3	2.6	29	30
		0.3	0	36	33	22	9	0.45	1.1	2.4	15	18
	300	5	0	27	22	36	15	1.04	1.2	2.4	28	35
		0.3	0	28	26	32	14	0.85	1.1	2.3	24	30
**7**	360	5	35	39	33	15	13	0.39	1.2	1.2	26	−1
		5	25	39	33	15	14	0.40	1.2	1.1	33	4
		5	15	37	35	15	13	0.39	1.1	1.2	28	−5
		5	0	37	34	14	14	0.39	1.1	1.0	37	−4
	300	5	0	39	30	14	17	0.45	1.3	0.8	32	−5

^a^Irradiated in pH 9 aqueous buffer at 0–35 °C with a Xenon lamp fitted with a band-pass filter; [AC] = 0.4 mM (fixed); [CD] = 0.12 or 2 mM. ^b^Irradiation wavelength. ^c^Host–guest ratio. ^d^Relative yield and ee determined by chiral HPLC on a tandem column of Intersil ODS-2 (GL Science) and Chiralcel OJ-R (Daicel); error in ee: ±0.7%. ^e^(**3**+**4**)/(**1**+**2**). ^f^Positive/negative ee sign corresponds to the excess of the first/second-eluted enantiomer, respectively. ^g^Ref [12]. ^h^Ref [20].

Closer examination of the product distributions revealed the contrasting behavior of the *anti/syn* ratio in HT versus HH dimers. As can be seen from the *anti/syn* ratios for the HT and HH dimers shown in Table 1, the modifications of γ-CD only slightly alter the **1**/**2** ratio from 0.9 for native γ-CD to 1.1–1.4 for **5**–**7**. In sharp contrast, the **3**/**4** ratio was more susceptible to rim modification, in particular rigid capping was enhanced from 1.2 for native γ-CD to 1.5 for biphenyl-capped **5** and even to 2.3–3.3 for naphthalene-capped **6**, but was practically unaffected at 1.0–1.2 for naphthalene-appended **7**. These results are quite reasonable, as the electrostatic repulsion of the carboxylate anions of two ACs in the CD cavity should be stronger in an HH-oriented complex than in an HT-oriented one due to the shorter inter-anion distance in the former. In summary, naphthalene-capping greatly enhances the formation of HH, in particular *anti*-HH, dimers as a combined effect of increased hydrophobicity and electrostatic repulsion.

The hydrophobic modifications at the primary rim reduced the ee of chiral HT dimer **2**, suggesting that the achiral substituent introduced altered the chiral environment of γ-CD cavity. In this context, it is interesting to note that naphthalene-capped **6** and naphthalene-appended **7** give **2** in 29% and 37% ee, respectively, which are only slightly smaller than that obtained with native γ-CD (41% ee), whereas biphenyl-capped **5** gave antipodal **2** in −58% ee under comparable conditions (Table 1). Molecular model examinations indicated that the biphenyl group, attached to the A and D glucose units of γ-CD **5**, covers half of the primary rim. In the HT-oriented precursor complex of AC with **5**, one of the carboxylate groups is inevitably exposed to the bulk water through the narrowed primary rim, leading to a highly restricted conformation, which significantly differs from the original one achieved in the native γ-CD cavity. In contrast, the naphthalene moiety is anchored to only one glucose unit in **7** or to the A and C glucose units in **6**, leaving a larger opening for the carboxylate tail of the AC and more freedom for the HT-oriented AC pair in the cavity, a situation similar to native γ-CD. Based on these considerations, we may conclude that the steric restriction caused by the capping group, rather than its rigidity, is the real cause of the chirality switching observed for **5**.

Interestingly, the use of naphthalene-capped **6** greatly improved the chemical and optical yields of **3**, while native γ-CD, biphenyl-capped **5**, and naphthalene-appended **7** afforded almost racemic or antipodal **3** in much smaller chemical and optical yields. The HH-oriented AC pair in a complex precursor to **3** is likely to conceal the hydrophobic anthracene moiety inside the cavity with the carboxylate groups being exposed to the bulk water near the secondary rim of γ-CD. The enhanced chemical and optical yields observed for **3** should reflect the totally altered chiral environment, which favors the formation of one of the diastereomeric HH-oriented precursor complexes in the modified CD cavity of **6**. By lowering the temperature to 0 °C, the system was optimized to give *anti*-HH **3** in 34% yield with an ee of 30%, which is much higher than the corresponding values obtained with native or any other capped γ-CDs so far examined in aqueous solutions [20,22].

We further examined indirect FRET excitation (intramolecular photosensitization) of AC included in host **6**. Mechanistically, FRET excitation of AC is highly advantageous from the photochirogenic point of view, since the static energy transfer to an AC molecule accommodated in the CD cavity is much faster and more efficient than the dynamic energy transfer to an AC in bulk solution (even if it exists), thus minimizing the unfavorable achiral “outside” photoreaction. Irradiation of an aqueous solution containing 0.4 mM AC and 2 mM **6** was performed at 300 nm, where 98.5% of the incident light was absorbed by the naphthalene moiety of **6** and therefore it is possible to examine the effects of FRET excitation on the distribution and ee of cyclodimers. As can be seen from Table 1, FRET excitation at 300 nm gave **3** in 36% yield and 35% ee, both of which are appreciably higher than the corresponding values obtained upon direct AC excitation at 360 nm. The enhanced stereoselectivity is attributed to the smaller contribution of the photoreaction outside the CD cavity, where the HT dimers are favored and chiral **2** and **3** produced should be racemic. To the best of our knowledge, this is the first example of FRET sensitization applied to photochirogenesis.

In supramolecular photochirogenesis, an excess amount of the chiral host is often the prerequisite for minimizing contamination from undesirable racemic photoreactions occurring outside chiral host. In the present system, it is likely that only AC that is included in the CD cavity can be FRET-sensitized by naphthalene, since the FRET efficiency is inversely proportional to the sixth power of donor–acceptor distance. We expected therefore that the AC photocyclodimerization could be catalyzed even with a sub-equivalent amount of the chiral FRET host. Indeed, the FRET-sensitized photocyclodimerization of AC with 0.3 equiv of **6** gave dimer **3** in 32% yield and 30% ee, which are only slightly decreased from the original 36% yield and 35% ee obtained with 5 equiv of **6** (Table 1). In contrast, the chemical yield of **3** was reduced from 34% to 22% and the ee from 30% to 18% upon direct AC excitation at 360 nm.

## Conclusion

In the present work, naphthalene-appended and -capped γ-CDs were synthesized to investigate the effects of naphthalene capping and of FRET excitation on the complexation and supramolecular photochirogenic behavior of AC. Compared to native γ-CD, the naphthalene-modified γ-CDs showed 3.5–5.8 fold larger first binding constants and 2.5–3.0 fold smaller second binding constants, which resulted in roughly two-fold larger overall affinities, due to increased cavity hydrophobicity. Fluorescence spectral examination revealed that the FRET from the excited naphthalene on CD rim to AC included in the CD cavity is operative upon excitation of the naphthalene chromophore at 296 nm. Circular dichroism spectral studies revealed that the two AC molecules are arranged in a right-handed screw sense in the CD cavity. Direct excitation at 360 nm of AC accommodated in the cavity of modified γ-CD afforded HH cyclodimers (despite electrostatic repulsion between the HH-oriented carboxylate anions of the AC) in combined yields of up to 51%, which is much higher than that (11%) obtained with native γ-CD. In particular, both the chemical and optical yields of HH dimer **3** were significantly enhanced from 6% to 34% and from −1% ee to 30% ee by introducing a naphthalene-cap to γ-CD. More interestingly, FRET sensitization by exciting the naphthalene-cap of **6** at 300 nm afforded HH dimer **3** in a further enhanced yield of 36% with an ee of 35%. In the FRET sensitization**,** the high stereoselectivity was maintained even when the host/guest ratio was reduced to 0.3, thus achieving catalytic supramolecular photochirogenesis.

## Experimental

**General**. FAB mass spectra were measured on a JEOL JMS-DX303 mass spectrometer. NMR spectra were recorded on a Bruker DRX-600 or a JEOL JNM-EX 400 spectrometer. UV–vis, fluorescence, and circular dichroism spectra were recorded in a UNISOKU USP-203CD cryostat with a JASCO V-560 spectrophotometer, JASCO FP-6500 luminescence spectrometer, and JASCO J-810 spectropolarimeter, respectively. Photoirradiation was performed in a UNISOKU USP-203 cryostat with an appropriate interference filter for 300 nm or 360 nm. Irradiated samples were subjected to chiral HPLC analysis on a tandem column of Intersil ODS-2(GL Science) and Chiralcel OJ-R (Daicel) with a 36:64 mixture of acetonitrile and water as eluent [12].

**Syntheses of modified γ-CDs 6 and 7.** For **6**: 6^A^,6^C^-ditosyl-γ-CD (321 mg, 0.2 mmol) and disodium 2,6-naphthalenedicarboxylate (52 mg, 0.2 mmol) were dissolved in 10 mL DMSO, and the mixture heated at 90 °C for 3 d. After cooling, the solution was concentrated to 0.5 mL under vacuum, trifluoroacetic acid added and the solution added dropwise to 30 mL acetone to give a precipitate. The precipitate was collected by filtration and purified by reverse-phase chromatography to give pure **6** (15.3 mg, 5.2% yield). ^1^H NMR (400 MHz, D_2_O): δ 8.14 (1H, s), 8.02 (1H, s), 7.78 (1H, d, *J* = 8.4 Hz), 7.69 (1H, d, *J* = 8.6 Hz), 7.62 (2H, d, *J* = 8.6 Hz), 4.97 (7H, m), 4.88 (2H, m), 4.79 (2H, m), 4.39 (2H, m), 4.31 (1H, m,), 4.18 (1H, m), 3.99–3.64 (13H, m), 3.63–3.31 (19H, m), 3.13 (4H, m), 2.56 (1H, d, *J* = 11.0 Hz,), 2.39 (1H, d, *J* = 11.1 Hz,), 2.27 (1H, d, *J* = 11.2 Hz,), 2.21 (1H, m), 1.60 (1H, br). ^13^C NMR (150 MHz, D_2_O): δ 167.85, 166.52, 133.85, 133.41, 132.71, 132.25, 131.98, 129.86, 129.50, 128.72, 125.94, 125.38, 102.36, 102.01, 101.84, 101.75, 101.67, 101.53, 81.21, 80.70, 80.07, 79.74, 74.86, 73.57, 73.26, 72.99, 72.81, 72.69, 72.61, 72.49, 72.31, 72.06, 71.94, 71.83, 71.68, 71.53, 71.22, 70.55, 68.13, 66.09, 60.23, 60.04, 59.91, 59.83, 58.52. HR–FAB-MS: Calc. for [**6**+Na]^+^, C_60_H_86_NaO_42_ 1499.43, found: 1499.43.

For **7**: 2,6-naphthalenedicarboxylic acid (216 mg, 1 mmol) and γ-CD (1296 mg, 1 mmol) were dissolved in 10 mL DMF, to which DCC (310 mg, 1.5 mmol) and HOBT (54 mg, 0.4 mmol) were added and the solution stirred at room temperature for 2 d. The reaction mixture was poured into 150 mL dry acetone to give a white precipitate. The precipitate was collected by filtration, dissolved in 10% aqueous methanol and then subjected to reverse–phase HPLC separation to give **7** (194 mg, 13% yield). ^1^H NMR (600 MHz, 4:1 DMSO-*d*_6_–D_2_O): δ 8.58 (1H, d, *J* = 12.4 Hz,), 8.54 (1H, s), 8.13 (1H, dd, *J* = 8.5, 2.6 Hz,), 8.10 (1H, d, *J* = 8.5 Hz,), 7.99 (1H, d, *J* = 8.8 Hz), 7.96 (1H, d, *J* = 8.6 Hz,), 4.93 (1H, d, *J* = 3.3 Hz), 4.89 (1H, d, *J* = 3.3 Hz), 4.83 (6H, m), 4.64 (1H, m), 4.36 (1H, m), 3.66–3.52 (24H, m), 3.52–3.39 (14H, m), 3.38–3.26 (18H, m). ^13^C NMR (150 MHz, 4:1 DMSO-*d*_6_–D_2_O) δ 167.71, 166.09, 149.51, 137.12, 134.64, 130.64, 130.49, 129.11, 126.42, 126.01, 124.42, 102.84, 102.01, 101.81, 82.30, 81.25, 81.17, 81.12, 81.04, 80.73, 73.30, 73.07, 72.97, 72.70, 72.47, 72.39, 69.46, 64.89, 60.22, 60.08, 59.89. HR–FAB-MS: Calc. for [**7**+Na]^+^, C_60_H_86_NaO_43_ 1517.44, found: 1517.45.

## Supporting Information

File 1 NMR and HR–MS data of compounds **6** and **7**.
