# Supplementary material for: Supramolecular FRET photocyclodimerization of anthracenecarboxylate with naphthalene-capped γ-cyclodextrin
Source: Beilstein J Org Chem. 2011 Mar 7;7:290–7. doi: 10.3762/bjoc.7.38 (PMC3062990; doi:10.3762/bjoc.7.38)
Supplement: File 1 — NMR and HR–MS data of compounds 6 and 7. [file Beilstein_J_Org_Chem-07-290-s001.pdf]

Supporting Information

for

Supramolecular FRET photocyclodimerization of anthracenecarboxylate with naphthalene-capped γ-cyclodextrin

Qian Wang[1,2], Cheng Yang*[1], Gaku Fukuhara[1], Tadashi Mori[1], Yu Liu[2], and Yoshihisa Inoue*[1]

Address: [1]Department of Applied Chemistry, Osaka University, 2-1 Yamada-oka, Suita 565-0871, Japan and [2]Department of Chemistry and State Key Laboratory of Elemento-Organic Chemistry, Nankai University Tianjin, 300071 (China)

Email: Cheng Yang - c.yang@chem.eng.osaka-u.ac.jp;
Yoshihisa Inoue - inoue@chem.eng.osaka-u.ac.jp

*****Corresponding author

NMR and HR–MS data of compounds 6 and 7.

Contents

General S3

Figure S1: ^{1}H NMR of **6** in D_2O at 20 °C S4

Figure S2: ^{13}C NMR of **6** in D_2O at 20 °C S5

Figure S3: ^{1}H NMR of **7** in 4:1 DMSO-d_6-D_2O at 20 °C S6

Figure S4: ^{13}C NMR of **7** in 4:1 DMSO-d_6-D_2O at 20 °C S7

Figure S5: HR–MS of **6**. S8

Figure S6: HR–MS of **7** S9

General

Materials. γ-CD (Aldrich) and other reagents (Tokyo Chemical Industry) were used without further purification.

Instruments. FAB mass spectra were measured on a JEOL JMS-DX303 mass spectrometer. NMR spectra were recorded on a Bruker DRX-600 or a JEOL JNM-EX 400 spectrometer. UV–vis, fluorescence, and circular dichroism spectra were recorded in a UNISOKU USP-203CD cryostat with a JASCO V-560 spectrophotometer, JASCO FP-6500 spectrofluorimeter, and JASCO J-810 spectropolarimeter, respectively.

Photolysis. Photoirradiations were performed in a UNISOKU USP-203 cryostat with an appropriate interference filter for 300 nm or 360 nm. Irradiated samples were subjected to chiral HPLC analysis on a tandem column of Intersil ODS-2(GL Science) and Chiralcel OJ-R (Daicel) with a 36:64 mixture of acetonitrile and water as eluent.

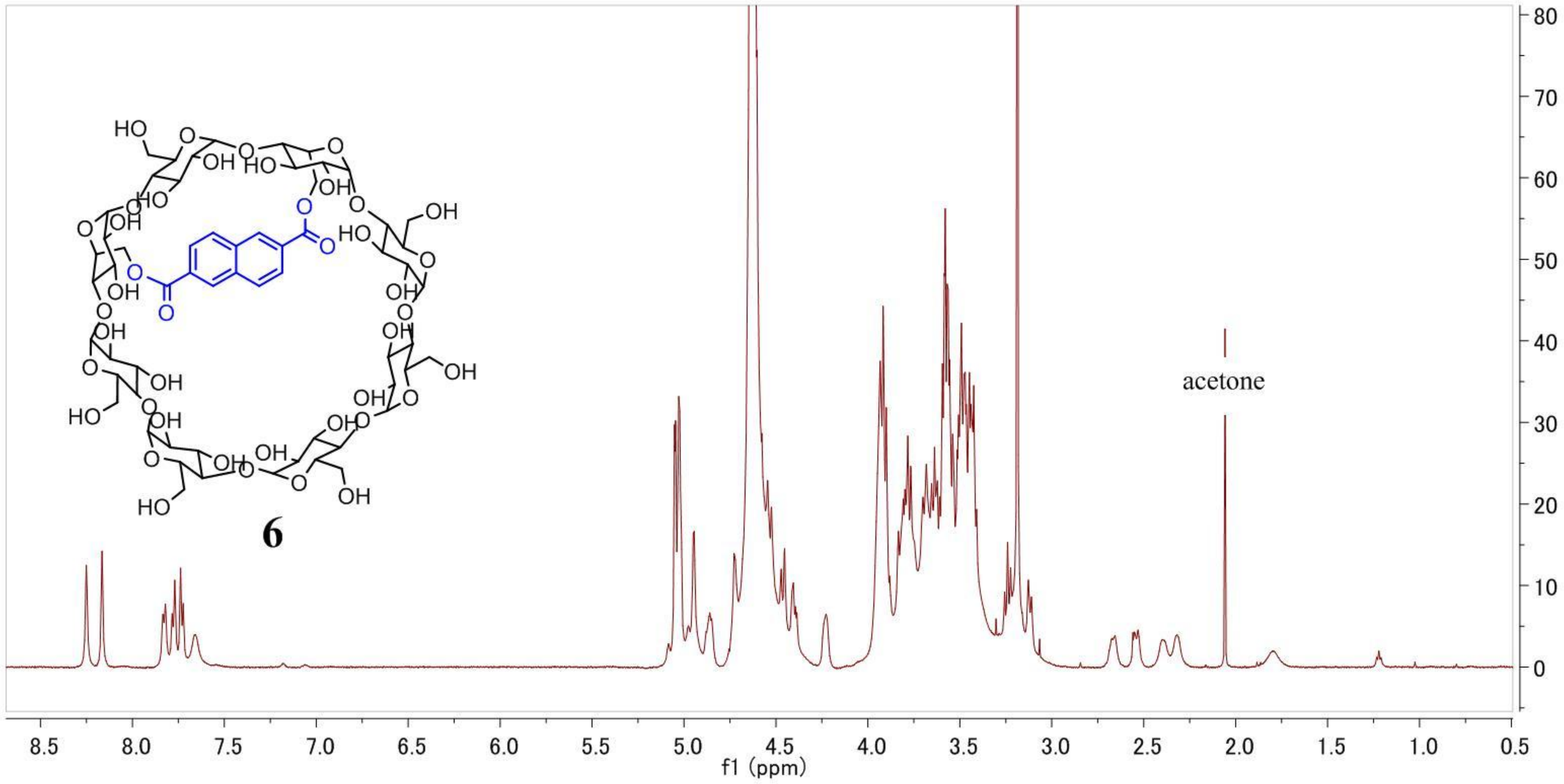

Figure S1: ^{1}H NMR of **6** in D_2O at 20 °C.

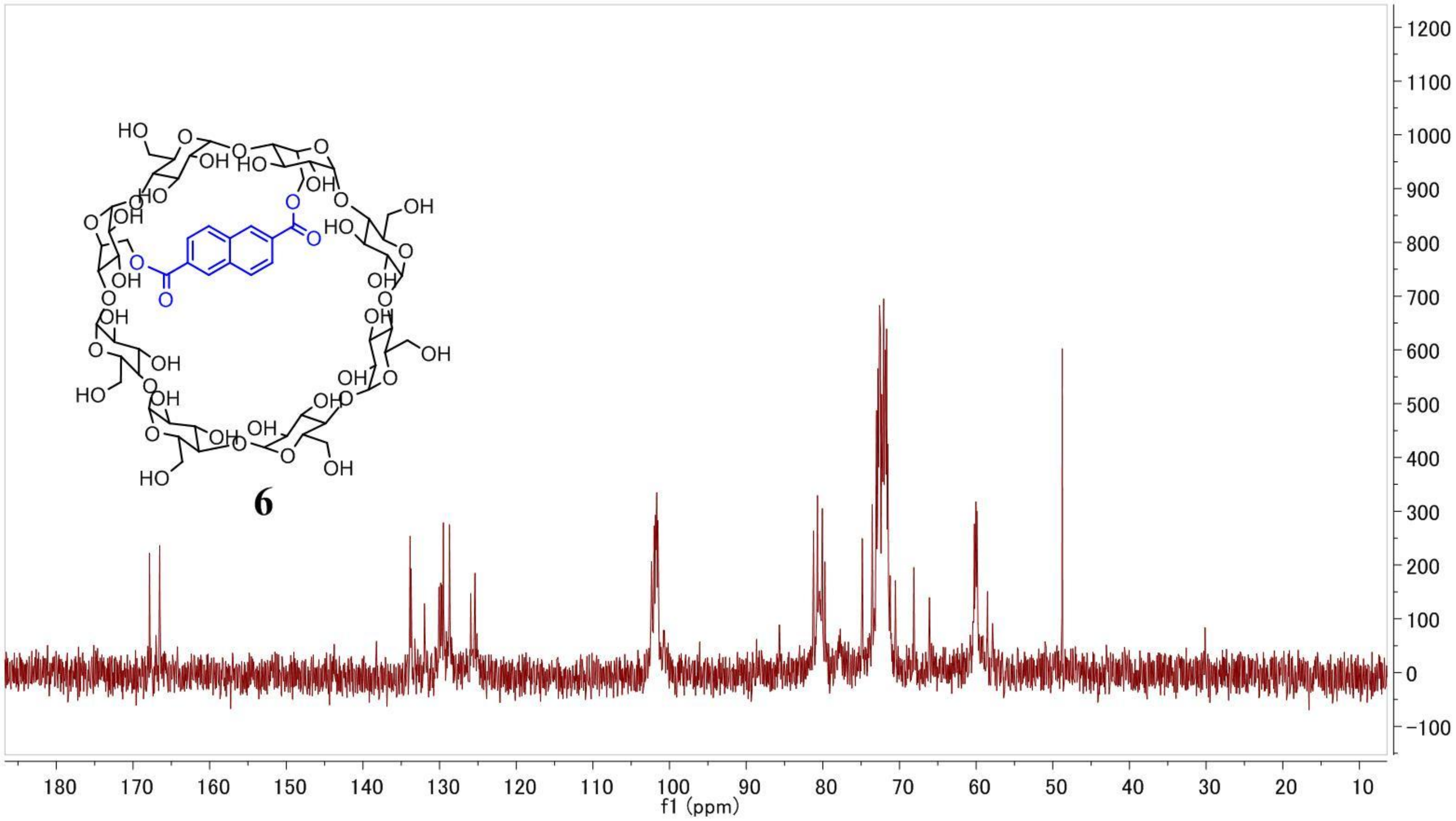

Figure S2: ^{13}C NMR of **6** in D_2O at 20 °C.

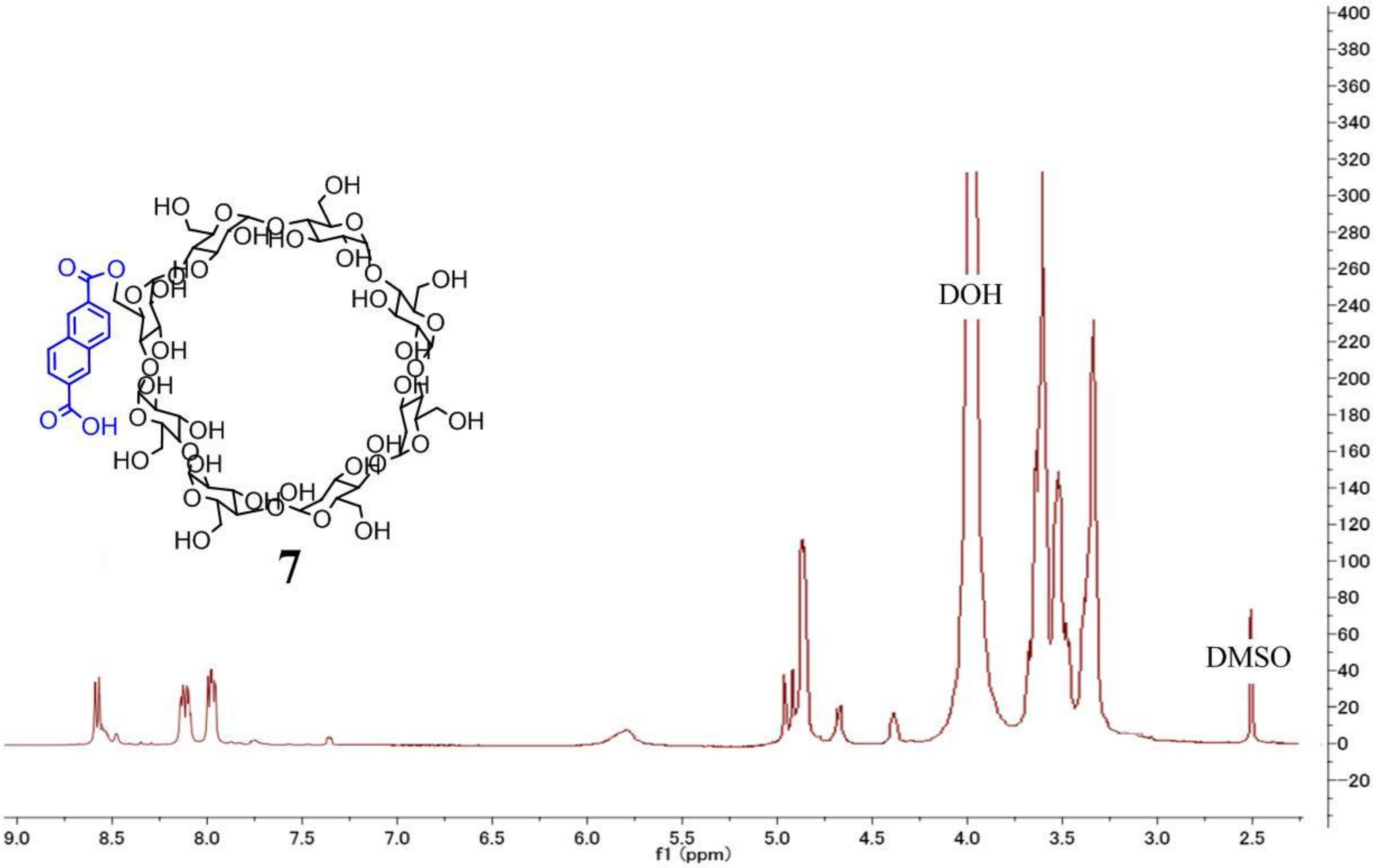

Figure S3: ^{1}H NMR of **7** in 4:1 DMSO-d_6-D_2O at 20 °C.

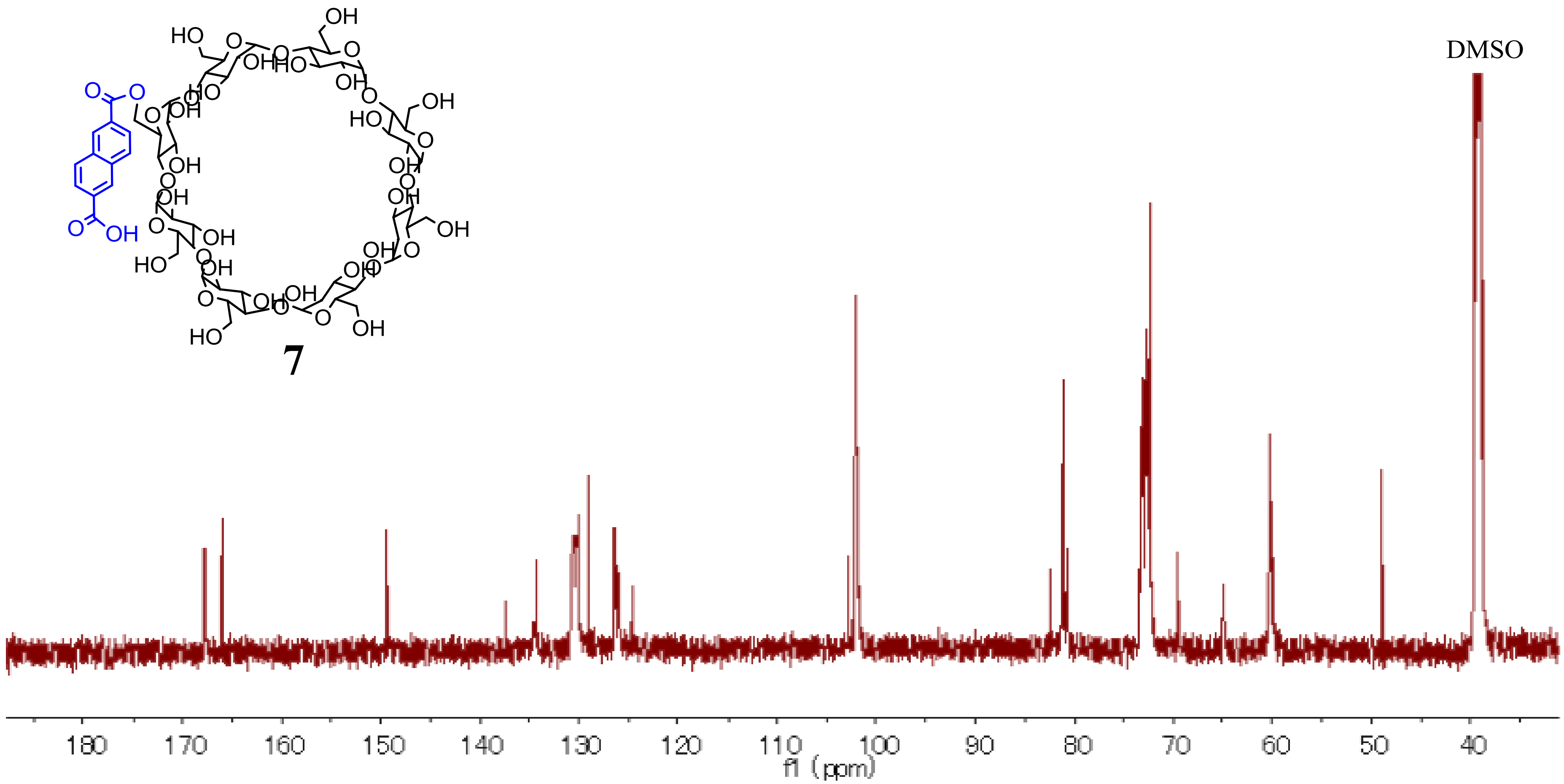

Figure S4: ^{13}C NMR of **7** in 4:1 DMSO-d_6-D_2O at 20 °C.

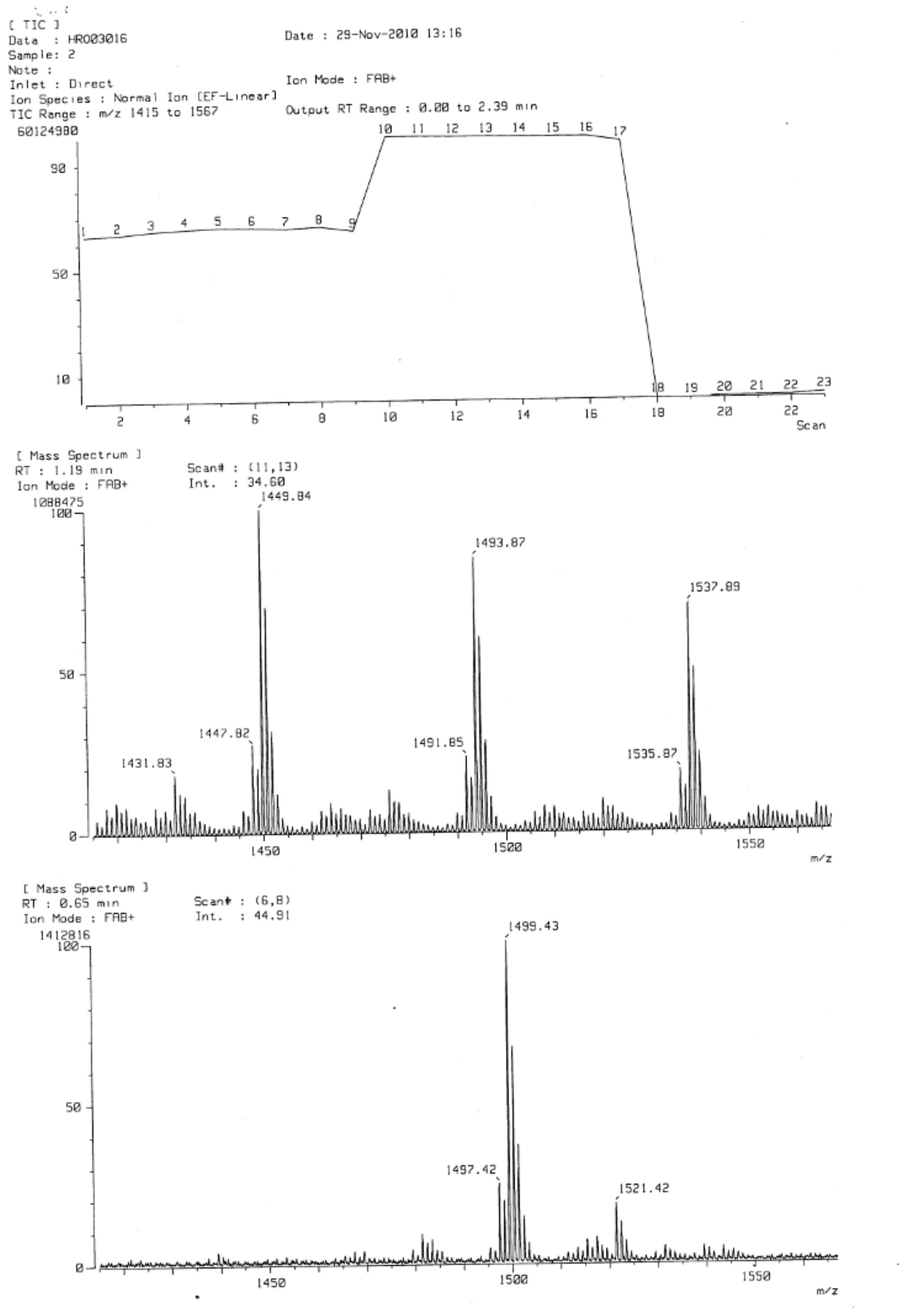

Pa

[Elemental Composition]
Data : HRO03016
Sample: 2 +Na]
Note :
Inlet : Direct
RT : 0.65 min
Elements : C 100/0, H 120/0, O 43/0, Na 1/0
Mass Tolerance : 10mmu
Unsaturation (U.S.) : -1.0 - 20.0

Date : 29-Nov-2010 13:16
Ion Mode : FAB+
Scan#: (6,8)

Observed m/z	Int%	Err[ppm / mmu]	U.S.	Composition
1499.4318	100.0	-1.2 / -1.7	18.5	C 60 H 84 O 42 Na

Figure S5: HR–MS of **6**. Calc. for [**6**+Na]$^+$, $C_{60}H_{86}NaO_{42}$ 1499.43, found: 1499.43.

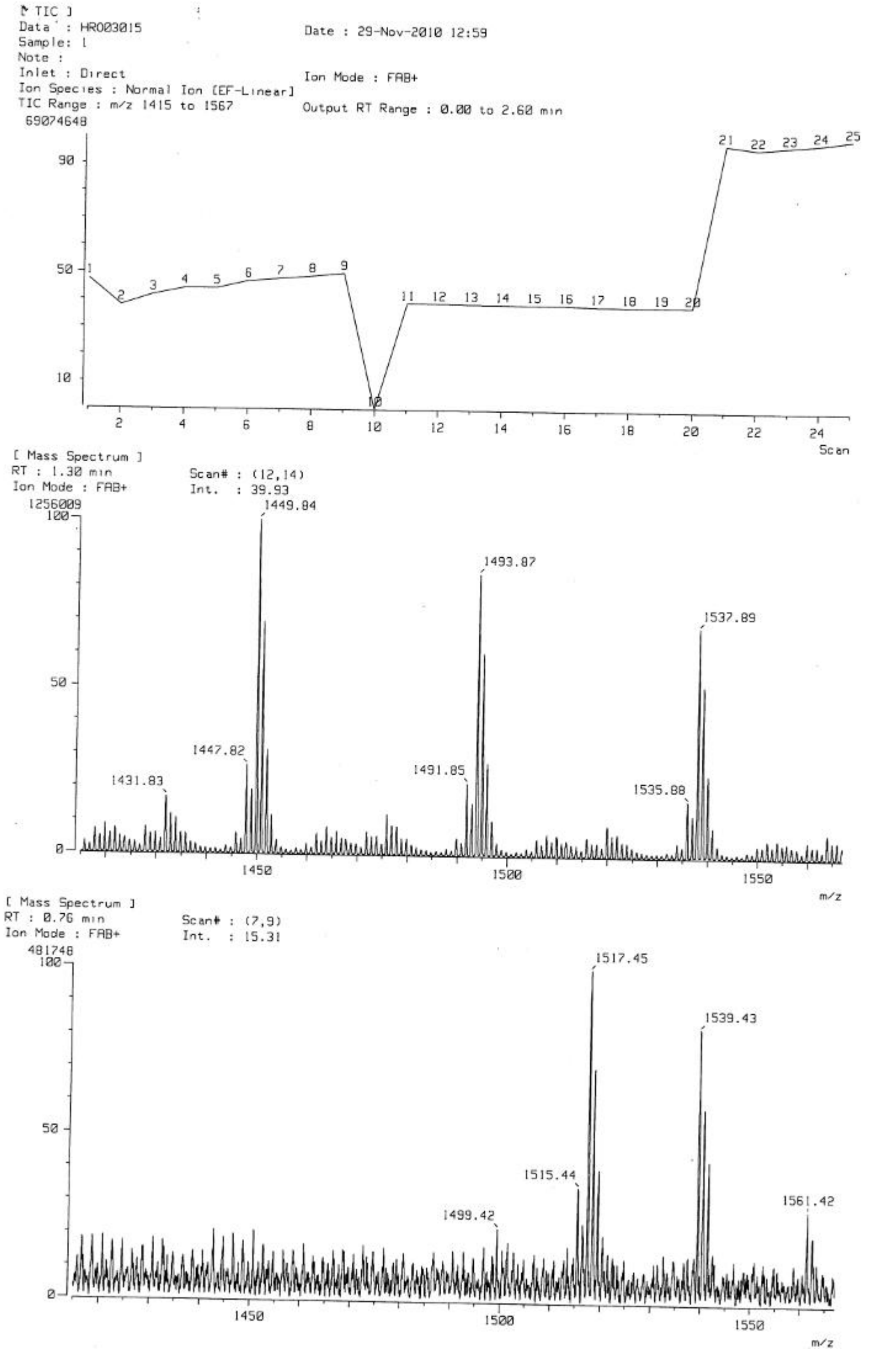

[Elemental Composition]

Page:

Data : HRO03015   Date : 29-Nov-2010 12:59
Sample: 1   +Na]
Note :
Inlet : Direct   Ion Mode : FAB+
RT : 0.76 min   Scan#: (7,9)
Elements : C 100/0, H 120/0, O 43/0, Na 1/0
Mass Tolerance : 10mmu
Unsaturation (U.S.) : -1.0 - 20.0

Observed m/z	Int%	Err[ppm / mmu]	U.S.	Composition
1517.4495	100.0	+3.6 / +5.4	17.5	C 60 H 86 O 43 Na

Figure S6: HR–MS of **7**. Calc. for [**7**+Na]$^+$, $C_{60}H_{86}NaO_{43}$ 1517.44, found: 1517.45.
